# Supplementary material for: Transcriptomic and Metabolomic Analyses of Soybean Protein Isolate on Monascus Pigments and Monacolin K Production
Source: J Fungi (Basel). 2024 Jul 19;10(7):500. doi: 10.3390/jof10070500 (PMC11277953; doi:10.3390/jof10070500)
Supplement: Supplementary file 1 [file jof-10-00500-s001.zip › jof-3090919-supplementary.pdf]

Supplementary Data

Table S1. HPLC gradient elution program for free amino acids.

T (min)	A% (Sodium acetate)	B% (Pure water)	C% (Acetonitrile)
0.0	84.0	8.0	8.0
0.3	84.0	8.0	8.0
4.0	69.0	15.5	15.5
9.5	75.0	12.5	18.0
17.0	45.0	27.5	27.5
28.0	40.0	30.0	30.0
34.0	0.0	50.0	50.0
38.0	0.0	50.0	50.0
39.0	84.0	8.0	8.0
40.0	84.0	8.0	8.0

Table S2. Primers used for RT-qPCR analysis.

Primer name	Sequence (5'to3')	Product size (bp)
Gene-315043-F	GAAGTCTCCCAGATTGGCCC	113
Gene-315043-R	ATCTCACTTCTGCGGGCTAC	
Gene-213838-F	CCCGAGAAGAAGCCCAACTT	163
Gene-213838-R	CGGTATTAGTGTGGCCCTGG	
Gene-256816-F	CTGGCCGCTATCGAAGATGA	106
Gene-256816-R	GGAGGAGTTCCAGTTCGGAG	
Gene-52089-F	GGTAGCATCGTCATCGTCGG	110
Gene-52089-R	AGCTCCAAGGAAGCACCAC	
Gene-170694-F	AGCCGTATTCTCAACGACCC	192
Gene-170694-R	CACCTGGGTCTCTGTCAACC	
Gene-272292-F	AAGGAGATTGGTCAGTGGGC	108
Gene-272292-R	ACTCCCAGAGCACCCATACA	
Gene-99680-F	CTGGCTCTCTCGTTTCCTCC	154
Gene-99680-R	GGCCTTCATACCACCCTCAG	
Gene-87025-F	CACATTGGCCCTGGTGTGTA	163
Gene-87025-R	CAGCCTTGGTCTCCTTCCAC	
Gene-304829-F	TCCTGACGGACTACCTGGAC	122
Gene-304829-R	CATCGTTCCCTTCTCAGCCA	
Gene-194396-F	TCCATAGCATGTGGTGCCTG	116
Gene-194396-R	CCTGGATTGCCGTTCACTCT	
actin-F	TCGAGAAGATGATGGGCAGC	179
actin-R	GGCCTTGACTGGAGACTTCC	

Table S3. Liquid chromatography gradient program.

T (min)	A% (Formic acid aqueous)	B% (Formic acid acetonitrile)
0.0	98.0	2.0
0.25	98.0	2.0
10.0	2.0	98.0
13.0	2.0	98.0
13.1	98.0	2.0
15.0	98.0	2.0

Table S4. Changes in free amino acids content (mg/L) during *Monascus* fermentation.

Free amino acids	Fermentation time (d)			
	0		9	
	CK	SPI	CK	SPI
Aspartate	-	5.02±5.02*	37.53±1.86*	21.20±3.85*
Glutamate	5461.15±220.08	4230.89±6.34*	4174.23±61.64	253.11±72.09*
Serine	-	-	-	-
Arginine	-	-	-	1.18±1.18

Glycine	-	-	-	0.51±0.51
Threonine	-	-	-	-
Proline	-	-	-	-
Alanine	5.20±0.26	2.71±2.71	9.88±0.63	31.86±5.36
Valine	118.00±1.43	116.77±0.80	107.36±1.06	107.38±1.86
Methionine	-	-	-	-
Cysteine	-	-	-	4.47±1.67
Isoleucine	-	-	-	-
Leucine	-	-	16.32±1.92*	37.14±2.95
Phenylalanine	-	-	-	-
Histidine	-	-	-	-
Lysine	-	-	-	-
Tyrosine	-	11.73±1.62*	-	1.11±1.11*
Total amino acids	5584.36	4367.12	4345.32	457.97

The results were expressed as the mean ± standard deviation, *$p < 0.05$.

Table S5. Transcriptome data statistics table.

Sample	Clean reads	Clean bases	GC content (%)	Q20 (%)	Q30 (%)
C1	22155506	6636433828	51.77	98.4	93.52
C2	25790278	7723253750	51.75	98.61	94.38
C3	21050169	6302292682	51.7	98.36	93.87
S1	20737551	6210837462	52.48	98.12	92.5
S2	21609174	6470634756	52.42	98.65	94.52
S3	21635178	6479293572	52.37	98.57	94.66

Table S6. Annotated number of DEGs.

DEG Set	Total	COG	GO	KEGG	KOG	NR	Pfam	Swiss-Prot	eggNOG
C vs S	3745	1423	2786	2242	1559	3739	2535	1986	2851

Table S7. Statistical table of differential metabolites.

Group	Totol_num	Diff_num	Up_num	Down_num
CI vs SI	2731	1449	969	480
CE vs SE	2731	1818	1029	789

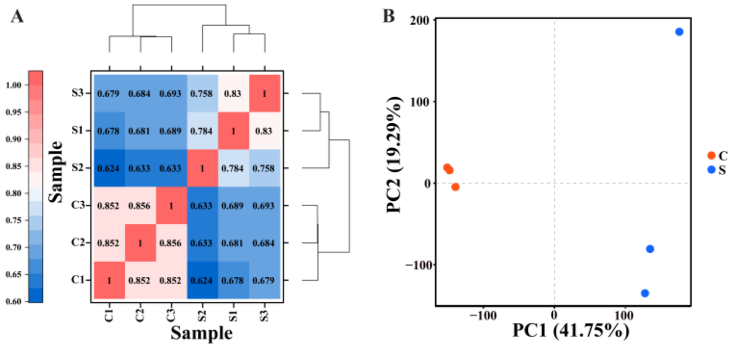

Figure S1. Sample correlation and principal component analysis. (**A**) Correlation heat map of samples. (**B**) principal component analysis (PCA) of samples.

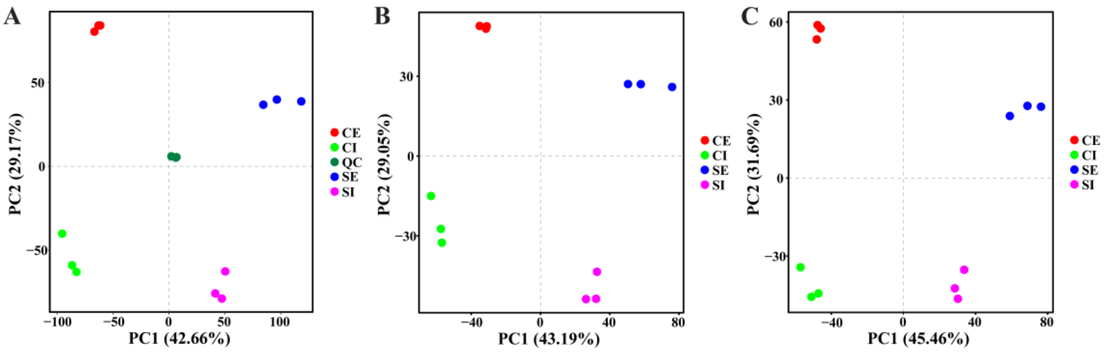

Figure S2. Principal component analysis. (**A**) Total PCA score plot; (**B**) positive ion mode; (**C**) negative ion mode.

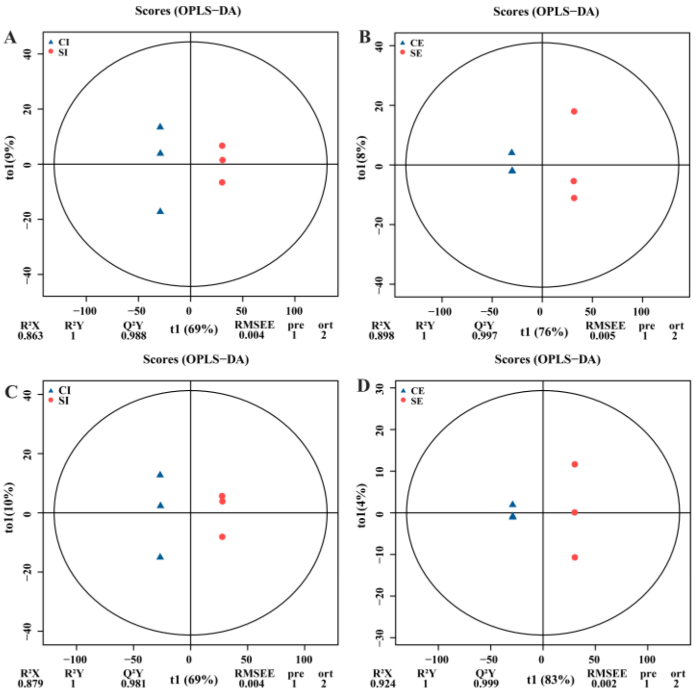

Figure S3. OPLS-DA permutation of control and SPI group under positive and negative mode. (**A**) Positive intracellular; (**B**) positive extracellular; (**C**) negative intracellular; (**D**) negative extracellular.

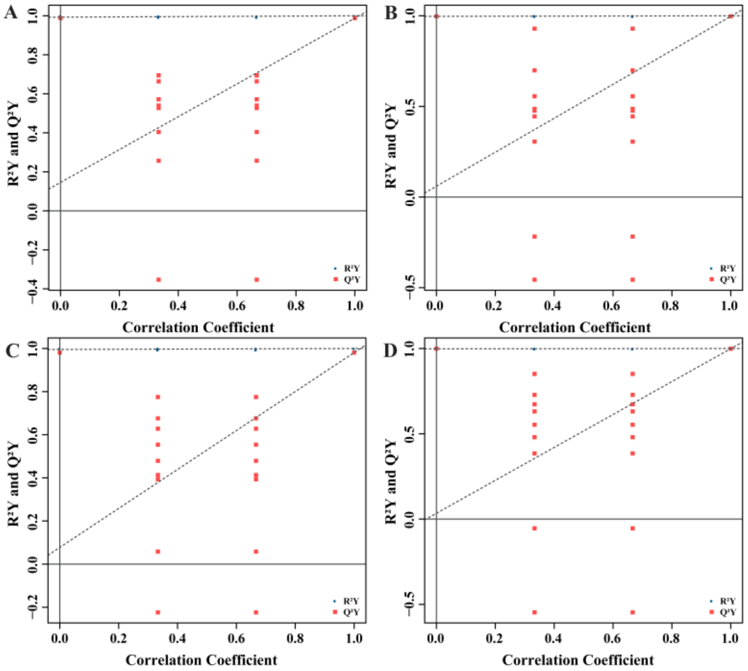

Figure S4. OPLS-DA permutation test of control and SPI group under positive and negative mode. (**A**) Positive intracellular; (**B**) positive extracellular; (**C**) negative intracellular; (**D**) negative extracellular.
